# Addressing racial disparities in surgical care with machine learning

**DOI:** 10.1038/s41746-022-00695-6

**Published:** 2022-09-30

**Authors:** John Halamka, Mohamad Bydon, Paul Cerrato, Anjali Bhagra

**Affiliations:** Mayo Clinic, Rochester, MN USA

**Keywords:** Health sciences, Health policy

## Abstract

There is ample evidence to demonstrate that discrimination against several population subgroups interferes with their ability to receive optimal surgical care. This bias can take many forms, including limited access to medical services, poor quality of care, and inadequate insurance coverage. While such inequalities will require numerous cultural, ethical, and sociological solutions, artificial intelligence-based algorithms may help address the problem by detecting bias in the data sets currently being used to make medical decisions. However, such AI-based solutions are only in early development. The purpose of this commentary is to serve as a call to action to encourage investigators and funding agencies to invest in the development of these digital tools.

Racial disparities in surgical care are a well-documented reality across the United States. Black patients receiving cardiac surgery have 17% and 26% higher odds of mortality and major postoperative complications, respectively, compared to White patients^1^. In spine surgery, the risk of postoperative complications has been estimated to be as much as 61% higher for Black patients. And it should be noted that these estimates were risk-adjusted for comorbidities, hospital characteristics, baseline patient status, and other factors, with the unadjusted discrepancies being even wider. The fact that Black patients receive a lower quality of care has been a known fact for over four decades^2–4^, but despite several efforts to address the problem^5–7^, the gap is far from closed^8^.

## Access to health care and barriers

One of the main reasons for the inequality in surgical outcomes is unequal access to health care. This lack of access has three main components: decreased exposure to preventive practices, lower rates of health care utilization, and delayed presentation. Black patients, for instance, are less likely to receive routine cancer screening and tend to present later for the management of preventable or early-detectable cancers, such as cervical or colorectal^9,10^. They are also less likely to receive hip and knee arthroplasty, lumbar surgery, carotid endarterectomy, and others^11^.

Although timely surgical intervention often results in better clinical outcomes, pathologies that are allowed to progress are less likely to respond to management. Several of the aforementioned factors—insurance, health literacy, economic status— may eventually result in Black patients presenting later in the natural timeline of their disease, restricting the benefit they may obtain from surgery. Among lung cancer patients of I–IIIA stage, for instance, Neroda et al. found that Black individuals were almost 70% more likely to receive delayed surgery—defined as a time from diagnosis to surgery of more than 6 weeks in this study. Similar findings have been reported in spine, benign brain tumor, and hip replacement surgery, with the delayed presentation being a mediator towards worse postoperative outcomes in Black patients^12–14^. Overall, hindered access to health care makes Black patients worse surgical candidates upon presentation.

It is no surprise to find that poor insurance coverage often contributes to the under-utilization of health care among Black individuals. Most studies supporting this observation have investigated the differences between private insurance, Medicare and Medicaid coverage, and lack of any insurance, and have found them to be significant. Black individuals are more prone to lose health care coverage at any point in their lives, recording a proportion of uninsured person-years of 0.20, compared to 0.12 among White individuals. Private insurance is associated with easier appointment scheduling^15^, delivery of more patient-friendly care practices— such as minimally invasive and outpatient surgery^16,17^—and superior surgical outcomes when compared to government payors and a lack of insurance coverage^18,19^. However, the comparison of private-payer programs, government-issued programs, and lack of insurance illuminates only part of the story, as there is a large heterogeneity among private insurance programs that could potentially impact access to health care. More specifically, higher-deductible plans discourage patients from pursuing contact with a provider and are more prevalent among Black individuals^20^.

Health literacy, prior individual experiences, and cultural traits may also contribute to the health disparities between Black and White individuals. Ibrahim et al. conducted a survey on patients with hip or knee osteoarthritis to assess the patients’ heuristics and expectations from care. They found that Black patients were more inclined towards complementary or self-administered therapeutic options, while they were less likely to consider joint replacement surgery^21^. Research also suggests that less education contributes to this phenomenon^22^. In addition, Black patients are more likely to overestimate the length of hospitalization, procedure-related pain, and disability; overall they are more skeptical about joint replacement surgery than White patients^23^.

## Comparing delivered quality of care

Another potential component of the racial disparities in surgical care is the discrepancies in the quality of care delivered. In a study by Rangrass et al. utilizing a national claims database, it was shown that hospital quality might explain as much as 35% of the observed discrepancy in mortality after coronary artery bypass graft surgery between Black and White patients^24^. However, this conclusion is refuted by Silber et al., who utilized the same database to study the same hypothesis on general surgical procedures. The investigators of the latter study found that discrepancies in outcomes were eliminated following matching Black and White patients on preoperative status; hence, they suggested that racial disparities should be attributed to the delayed access to care rather than the heterogeneity of care quality among providers and institutions^25^.

## Addressing surgical disparities

Several federal programs and scientific community initiatives have been launched to address racial disparities in health care during the past two decades^5–7^. In 2011, the Department of Health and Human Services announced a multidimensional plan to address the racial gap in healthcare: this plan included policy modifications, funding redistribution, and rewards for the care of socially disadvantaged populations, among others. Subsequently, the Affordable Care Act provided poorer individuals with enhanced insurance options. Buchmueller et al. found the Affordable Care Act to lower the uninsured rate among Black individuals by almost 35%. Nevertheless, overall, these measures have not been as impactful as desired, and the landscape remains essentially unchanged. In a study using the National Inpatient Sample, Best et al.^8^ investigated the utilization of nine common procedures by race relative to the proportion of races in the population and found the gap between them having become smaller in some cases, while larger in others, while at no point reaching equality between Black and White individuals. It is clear that racial disparities are still an unresolved problem in society, and the high-level policies employed so far have not proven sufficient to eradicate them.

## Can surgical bias yield to AI-based algorithms?

It is unrealistic to imagine that discrimination against various population subgroups can be resolved with artificial intelligence alone. The cultural, ethical, and sociological issues are far too complex to solve with digital tools, regardless of how sophisticated they may be. Nonetheless, AI and machine learning that addresses a variety of technological touch points can improve the profession’s ability to detect bias and improve patients’ access to surgical services and outcomes.

To ensure that all patients are ensured equal access to high-quality medical care, including surgical services, it is first necessary to analyze the data sets used to determine whether patients of color, women and those in lower socioeconomic groups are accurately represented in the data sets and algorithms used to determine the need for said services. As we have pointed out in a previous publication^26^, this has not always been the case. Obermeyer et al.’s^27^ analysis of a commercial database has demonstrated that, while Blacks were considerably sicker than White patients, based on signs and symptoms, the dataset did not recognize the greater disease burden in Blacks because it assigned risk scores based on total healthcare costs accrued. It is unrealistic to assume that such costs accurately measured patients’ needs; the lower cost among Blacks may have been due to less access to care, which in turn resulted from their distrust of the healthcare system and direct racial discrimination from providers^28^. Similar discrimination against women has been documented in medical imaging datasets used to train and test AI systems used for computer-assisted diagnosis^29^. There is also evidence to suggest that some machine learning enhanced algorithms that rely on electronic health record data under-represent patients in lower socioeconomic groups^30^.

Commercially available AI bias detection tools that have been used to help identify discrimination include concept activation vectors (TCAV), which are used by Google to measure bias by race, gender, and location^31^, and Audit-AI, which uses a Python library from Pymetrics that can detect discrimination by locating specific patterns in the training data^26,32^.

## Devising a comprehensive bias detection toolkit

While the aforementioned bias detection programs have merit, solving the problem of surgical bias will require a more comprehensive approach. That approach begins with a set of guidelines that set forth standards on how to conduct AI-related research and how to report it in the professional literature, including The Standard Protocol Items: Recommendations for Interventional Trials-Artificial Intelligence (SPIRIT-AI)extension, a set of guidelines designed to help researchers develop AI-related clinical trials^33^, and the Consolidated Standards of Reporting Trials-Artificial Intelligence (CONSORT-AI) extension^34^ Unfortunately, despite the recommendations from thought leaders regarding the importance of adhering to standards that would make algorithms more equitable, Lu et al. have found these guidelines are often ignored^35^.

They looked at 15 model reporting guidelines and reviewed 12 deployed Epic models. They found a median completion rate was 39% and stated: “…information on usefulness, reliability, transparency, and fairness was missing from at least half of documentation.”

Mayo Clinic is taking a more direct approach to algorithmic bias. Mayo Clinic Platform (MCP) has developed _Validate, a digital solution that helps measure model sensitivity, specificity, area under the curve (AUC), and bias, which in turn enables the system to break down the racial, gender, and socio-economic disparities in the delivery of care. Using the tool can lend credibility to models, accelerates adoption into clinical practice, and enables developers to more readily meet regulatory requirements for approval. It provides users with a series of descriptive statistics of model performance and data to demonstrate that the model was run against each demographic.

To illustrate _Validate’s performance, imagine that a developer wants to create a clinical solution that predicts whether a patient with signs and symptoms of appendicitis will need surgery or can be managed with antibiotics. Inputs fed into the algorithm might include all historical patient data, including demographics, prior diagnoses, a history of abdominal abnormalities, and family history of the same. _Validate would provide *testability* that has been missing from many commercially available products. It enables health care stakeholders to test an AI model against an extensive data set and evaluate the reasonableness and usefulness of the result. In addition to its ability to evaluate and certify the quality and accuracy of an AI model, _Validate protects the intellectual property of the model and its data, using state-of-the-art de-identification protocols. With the assistance of Diagnostic Robotics, a validation services provider, _Validate analyzes the model’s performance, generating a table that includes true negatives, false negatives, true positives, and false positives, from which sensitivity, specificity, AUC, and positive predictive and negative predictive values can be derived. It can also perform a biased evaluation that takes into account race, ethnicity, age, obesity, behavioral health, genetic history, gender, and socioeconomic status markers.

Johns Hopkins University investigators are also taking measures to solve the bias problem. Wang et al have developed an 11-question checklist to help assess the validation of predictive models^36^. Among the issues that the checklist asks algorithm developers to take into consideration:

- “Is the prediction target an appropriate proxy for patient health care outcomes or needs?”
- “Are there any modeling choices made that could lead to bias? For example, are there any dependencies between inputs and outcomes that could lead to discriminatory performance across groups?”
- “Was the data used to train the model representative of the population in the deployment environment?”
- “Do validation studies report and address performance differences between groups?”

Innovation depends upon a perfect storm of technology, policy, and culture. Machine learning techniques, including deep learning systems, are mathematically robust in 2022 and commercially supported by all cloud providers, so it is fair to say that technology is not a rate-limiting step. Policies for the guardrails and guidelines of the machine learning life cycle to reduce bias and monitor ongoing fairness and usefulness, on the other hand, are still a work in progress; Several of us have assembled a multi-stakeholder coalition (coalitionforhealthai.org) to provide the foundational implementation guides that may evolve into policy. Culture likewise will require additional focus. We must set a cultural expectation that machine learning in healthcare should only be deployed in production when equity is a design principle. Finally, we believe that machine learning is only one tool in our quiver to reduce racial disparities in surgery, but it can be rapidly deployed, locally optimized, and monitored for impact over time.

### Reporting summary

Further information on research design is available in the Nature Research Reporting Summary linked to this article.

## Supplementary information

Reporting Summary

## References

[1] Mehta RH (2016). Association of hospital and physician characteristics and care processes with racial disparities in procedural outcomes among contemporary patients undergoing coronary artery bypass grafting surgery. Circulation.

[2] Carlisle DM, Leake BD, Shapiro MF (1997). Racial and ethnic disparities in the use of cardiovascular procedures: associations with type of health insurance. Am. J. Public Health.

[3] Lucas FL, Stukel TA, Morris AM, Siewers AE, Birkmeyer JD (2006). Race and surgical mortality in the United States. Ann. Surg..

[4] Bombardier C, Fuchs VR, Lillard LA, Warner KE (1977). Socioeconomic factors affecting the utilization of surgical operations. N. Engl. J. Med..

[5] Movement Is Life Caucus. *Movement Is Life: a Catalyst for Change: Addressing Musculoskeletal Health Disparities* (Movement Is Life Caucus, accessed May 2022); https://www.movementislifecaucus.com/wp-content/uploads/Movement-Is-Life-A-Catalyst-For-Change-Proceedings-Report.pdf (2011).

[6] US Department of Health and Human Services. *HHS Action Plan to Reduce Racial and Ethnic Disparities: a Nation Free of Disparities in Health and Health Care* (US Department of Health and Human Services, accessed May 2022); https://www.minorityhealth.hhs.gov/assets/PDF/Update_HHS_Disparities_Dept-FY2020.pdf (2011).

[7] O’Connor MI, Lavernia CJ, Nelson CL (2011). AAOS/ORS/ABJS Musculoskeletal Healthcare Disparities Research Symposium: Editorial comment: a call to arms: eliminating musculoskeletal healthcare disparities. Clin. Orthop. Relat. Res..

[8] Best MJ, McFarland EG, Thakkar SC, Srikumaran U (2021). Racial disparities in the use of surgical procedures in the US. JAMA Surg..

[9] Johnson NL, Head KJ, Scott SF, Zimet GD (2020). Persistent disparities in cervical cancer screening uptake: knowledge and sociodemographic determinants of papanicolaou and human papillomavirus testing among women in the United States. Public Health Rep. (Washington, DC: 1974)..

[10] Burgess DJ (2011). Presence and correlates of racial disparities in adherence to colorectal cancer screening guidelines. J. Gen. Intern. Med..

[11] Jha AK, Fisher ES, Li Z, Orav EJ, Epstein AM (2005). Racial trends in the use of major procedures among the elderly. N. Engl. J. Med..

[12] Elsamadicy AA (2018). Influence of racial disparities on patient-reported satisfaction and short- and long-term perception of health status after elective lumbar spine surgery. J. Neurosurg.: Spine SPI.

[13] Anzalone CL, Glasgow AE, Van Gompel JJ, Carlson ML (2019). Racial differences in disease presentation and management of intracranial meningioma. J. Neurolog. Surg. Part B Skull Base.

[14] Nayar SK (2020). Racial disparity in time to surgery and complications for hip fracture patients. Clin. Orthop. Surg..

[15] Hsiang WR (2019). Medicaid patients have greater difficulty scheduling health care appointments compared with private insurance patients: a meta-analysis. Inquiry.

[16] Mooney J (2021). Minimally invasive versus open lumbar spinal fusion: a matched study investigating patient-reported and surgical outcomes. J. Neurosurg. Spine.

[17] Mooney, J. et al. Outpatient versus inpatient lumbar decompression surgery: a matched noninferiority study investigating clinical and patient-reported outcomes. *J. Neurosurg. Spine* 1–13. 10.3171/2022.3.SPINE211558 (2022). 10.3171/2022.3.SPINE211558 35523251

[18] Curry WT, Carter BS, Barker FG (2010). Racial, ethnic, and socioeconomic disparities in patient outcomes after craniotomy for tumor in adult patients in the United States, 1988–2004. Neurosurgery.

[19] LaPar DJ (2010). Primary payer status affects mortality for major surgical operations. Ann. Surg..

[20] Cole MB, Ellison JE, Trivedi AN (2020). Association between high-deductible health plans and disparities in access to care among cancer survivors. JAMA Netw. Open.

[21] Ibrahim SA, Siminoff LA, Burant CJ, Kwoh CK (2001). Variation in perceptions of treatment and self-care practices in elderly with osteoarthritis: a comparison between African American and white patient s. Arthritis Rheum..

[22] Chaudhry SI (2011). Racial disparities in health literacy and access to care among patients with heart failure. J. Card. Fail..

[23] Ibrahim SA, Siminoff LA, Burant CJ, Kwoh CK (2002). Differences in expectations of outcome mediate African American/white patient differences in “willingness” to consider joint replacement. Arthritis Rheum..

[24] Rangrass G, Ghaferi AA, Dimick JB (2014). Explaining racial disparities in outcomes after cardiac surgery: the role of hospital quality. JAMA Surg..

[25] Silber JH (2015). Examining causes of racial disparities in general surgical mortality: hospital quality versus patient risk. Med. Care.

[26] Cerrato P, Halamka J, Pencina M (2022). A proposal for developing a platform that evaluates algorithmic equity and accuracy. BMJ Health Care Inf..

[27] Obermeyer Z (2019). Dissecting racial bias in an algorithm used to manage the health of populations. Science.

[28] Ledford H (2019). Millions of black people affected by racial bias in health- care algorithms. Nature.

[29] Larrazabal AJ (2020). Gender imbalance in medical imaging datasets produces biased classifiers for computer-aided diagnosis. Proc. Natl Acad. Sci. USA.

[30] Gianfrancesco MA (2018). Potential biases in machine learning algorithms using electronic health record data. JAMA Intern. Med..

[31] Kim, B., Wattenberg, M. & Gilmer, G. Interpretability beyond feature attribution: quantitative testing with concept activation vectors (TCAV). In *Proc. 35th International Conference on Machine Learning*, (ed. Lawrence, N.) (Stockholm, Sweden, PMLR 80, MLR Press, 2018).

[32] Pymetrics/audit, AI. (Pymetrics/audit, AI, accessed May 2022) https://github.com/pymetrics/audit-ai (2020).

[33] Cruz Rivera S (2020). Guidelines for clinical trial protocols for interventions involving artificial intelligence: the SPIRIT-AI extension. Nat. Med..

[34] Liu X (2020). Reporting guidelines for clinical trial reports for interventions involving artificial intelligence: the CONSORT-AI extension. Nat. Med..

[35] Lu J (2022). Assessment of Adherence to Reporting Guidelines by Commonly Used Clinical Prediction Models From a Single Vendor: A Systematic Review. JAMA Netw Open.

[36] Wang HE (2022). A bias evaluation checklist for predictive models and its pilot application for 30-day hospital readmission models. J. Am. Med. Inform. Assoc..

